# Synthesis and Structural Characterization of Fluorinated Thiosemicarbazones

**DOI:** 10.3390/molecules181013111

**Published:** 2013-10-22

**Authors:** Juan L. Bautista, Marcos Flores-Alamo, Jorge Tiburcio, Rebeca Vieto, Hugo Torrens

**Affiliations:** 1 Facultad de Ciencias Químicas, Universidad Autónoma Benito Juárez de Oaxaca. Av. Universidad S/N Cinco Señores, Oaxaca de Juárez, Oaxaca 68000, Mexico; E-Mail: jlbautista@hotmail.com; 2 Facultad de Química, UNAM, Cd. Universitaria, 04510 México D.F., Mexico; E-Mails: mfa@unam.mx (M.F.A.); rebecaq@gmail.com (R.V.); 3 Departamento de Química, CINVESTAV, Av. Instituto Politécnico Nacional No. 2508, San Pedro Zacatenco, México 07360, Mexico; E-Mail: jtiburcio@cinvestav.mx

**Keywords:** fluorobenzaldehyde, sulfur, thiosemicarbazones, crystal structure

## Abstract

Six new fluorinated thiosemicarbazones R-C(R′)=N-NH-C(S)NH_2_ (R = 2,4-C_6_H_3_F_2_, R′ = H (**1)**; R = 2,5-C_6_H_3_F_2_, R′ = H (**2**); R = 2,6-C_6_H_3_F_2_, R′ = H (**3**); R = 3,4-C_6_H_3_F_2_, R′ = H (**4**); R = 3,5-C_6_H_3_F_2_, R′ = H (**5**) and R = 4-C_6_H_4_F, R′ = C_6_H_5_, (**6**)) have been prepared. The molecular structures of compounds **1** to **6** have been determined.

## 1. Introduction

Studies focusing on thiosemicarbazones date back to the beginning of the last century, but the first reports on their pharmaceutical applications as drugs against leprosy and tuberculosis were published in the 1950s [1,2]. Later, their antiviral and antitumoral [3] properties, as well as their involvement in the treatment of smallpox [4] triggered a huge amount of research.

Today, thiosemicarbazones are the focus of a growing interest because many of their properties are relevant to a wide range of fields. These compounds, formed by the condensation of thiosemicarbazide and aldehyde or ketone have endless combinations and the presence of at least amide, imine and thione groups, makes them a class of potent potential polydentate ligands that have proven to be very efficient metal chelators and it is not surprising that numerous thiosemicarbazone complexes have been prepared and characterized [5].

Studies of thiosemicarbazone derivatives include investigations of corrosion inhibitors for metal alloys [6], metal extraction and separation [7] applications in spectrophotometry, fluorometry, atomic absorption spectrophotometry and as chemical sensors [8], supramolecular chemistry [9], optoelectronics [10] and pharmacological activity [11] as antitumoral, fungicidal, bactericidal and antiviral drugs.

We have been interested in fluorinated compounds for a long time [12] since inclusion of fluorine atoms in molecules often has a marked effect on their physical and chemical properties. For example, although it has been recognized that fluorine-containing drugs are often far more therapeutically active than their non-fluorinated analogs [13] it is surprising that relatively very little is known about the properties of fluorine-containing thiosemicarbazones [14,15]. In this paper, we report the syntheses of six new polyfluorobenzaldehyde thiosemicarbazones: RC(R′)=N-NH-C(S)NH_2_(R = 2,4-C_6_H_3_F_2_, R′ = H (**1)**; R = 2,5-C_6_H_3_F_2_, R′ = H (**2**); R = 2,6-C_6_H_3_F_2_, R′ = H (**3**); R = 3,4-C_6_H_3_F_2_, R′ = H (**4**); R = 3,5-C_6_H_3_F_2_, R′ = H (**5**) and R = 4-C_6_H_4_F, R′ = C_6_H_5_, (**6**)) and their corresponding X-ray diffraction structures.

## 2. Results and Discussion

**Scheme 1:**
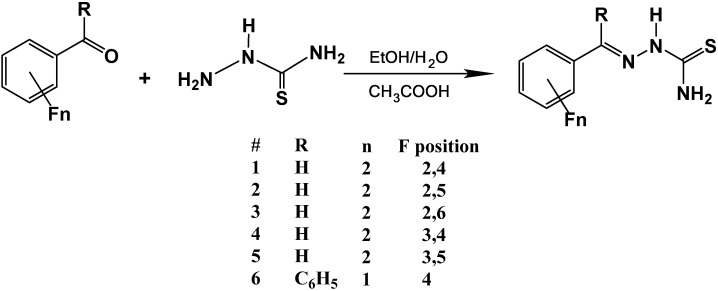
Preparation of the polyfluoroaryl thiosemicarbazones **1** to **6**.

The corresponding aldehydes RCHO (R = 2,4-C_6_H_3_F_2_, (**1**); R = 2,5-C_6_H_3_F_2_, (**2**); R = 2,6-C_6_H_3_F_2_, (**3**); R = 3,4-C_6_H_3_F_2_, (**4**); R = 3,5-C_6_H_3_F_2_, (**5**) or ketone ((4-C_6_H_4_F)C(=O)(C_6_H_5_) (**6**)) was dissolved in ethanol at room temperature and added to solutions of thiosemicarbazide in a 1:1 mixture of ethanol/water, along with catalytic amounts of acetic acid (Scheme 1). The reaction mixtures were refluxed for 24 h and then the solvent was reduced under vacuum to one third of the original volume, from which the products precipitated as white crystalline solids. Compounds **1**–**6** were recrystallized from ethanol/water (1:1) and characterized by ^1^H-, ^13^C- and ^19^F{^1^H}-NMR, MS and IR.

**Figure 1:**
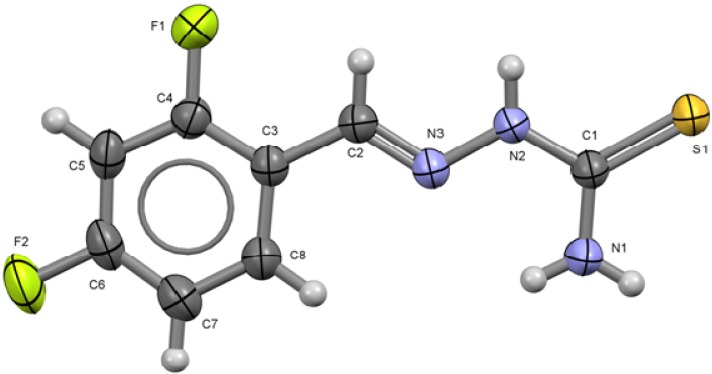
ORTEP drawing of 2,4-C_6_H_3_F_2_-CH=N-NH-C(S)NH_2_ (**1**). Thermal ellipsoids are shown at 50% of probability. Selected bond lengths (Å) and angles (°): S1-C1 1.690(16), C1-N1 1.315(2), C1-N2 1.3400(19), N2-N3 1.3692(17), N3-C2 1.2728(19) C2-C3 1.462(2) C4-F1 1.3565(18), C6-F2 1.361(2); S1-C1-N1 122.99(13), S1-C1-N2 119.97(11), N1-C1-N2 117.04(15), N2-N3-C2, N3-C2-C3 117.26(13).

The X-ray diffraction molecular and crystal structures of compounds **1** to **6** have been determined [16]. The molecular structures and atom numbering schemes of compounds **1** to **6** are shown on Figure 1 to Figure 6 along with selected bond distances and angles.

**Figure 2:**
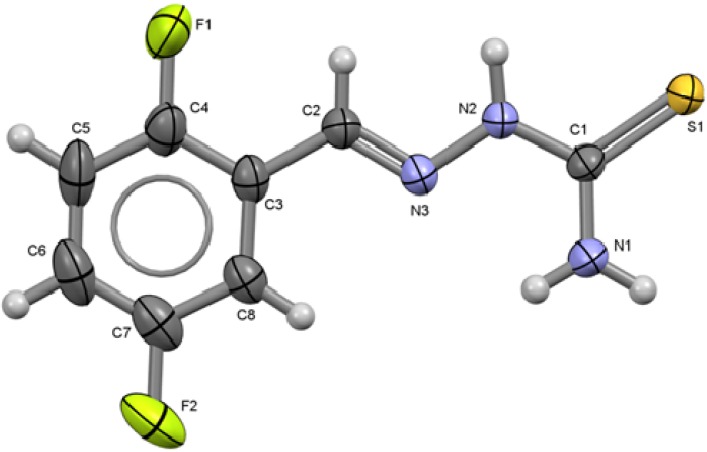
ORTEP drawing of 2,5-C_6_H_3_F_2_-CH=N-NH-C(S)NH_2_ (**2**). Thermal ellipsoids are shown at 50% of probability. Selected bond lengths (Å) and angles (°): S1-C1 1.6881(15), C1-N1 1.3182(19), C1-N2 1.3438(18), N2-N3 1.3752(17), N3-C2 1.2699(19) C2-C3 1.462(2) C4-F1 1.355(2), C7-F2 1.356(2); S1-C1-N1 123.45(12), S1-C1-N2 119.04(11), N1-C1-N2 117.51(14), N2-N3-C2 115.50(13), N3-C2-C3 120.02(15).

**Figure 3:**
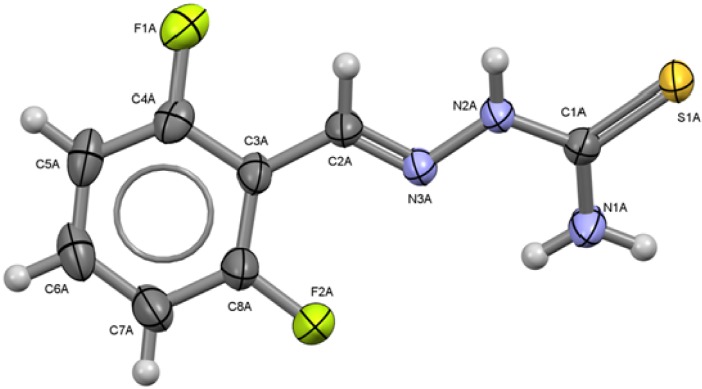
ORTEP drawing of 2,6-C_6_H_3_F_2_-CH=N-NH-C(S)NH_2_ (**3**, molecule A). Thermal ellipsoids are shown at 50% of probability. Selected bond lengths (Å) and angles (°): S1A-C1A 1.6926(18), C1A-N1A 1.320(2), C1A-N2A 1.339(2) N2A-N3A 1.378(2), N3A-C2A 1.276(2), C2A-C3A 1.458(3), C4A-F1A 1.348(2), C8A-F2A 1.341(2); S1A-C1A-N1A 123.39(14), S1A-C1A-N2A 118.68(14), N1A-C1A-N2A 117.92(17), N2A-N3A-C2A 114.48(15), N3A-C2A-C3A 122.99(17).

**Figure 4:**
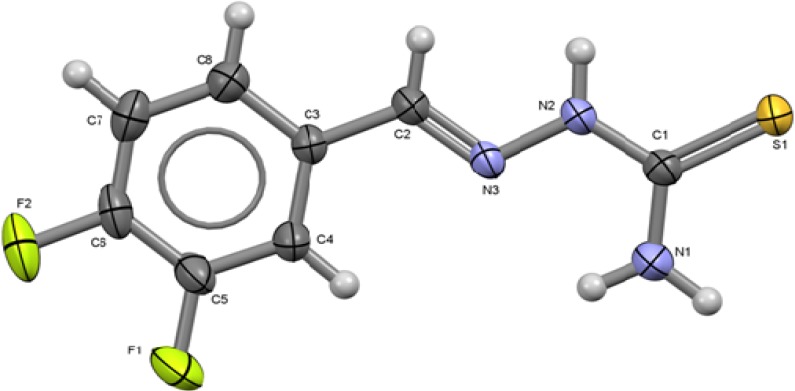
ORTEP drawing of 3,4-C_6_H_3_F_2_-CH=N-NH-C(S)NH_2_ (**4**). Thermal ellipsoids are shown at 50% of probability. Selected bond lengths (Å) and angles (°): S1-C1 1.6788(14), C1-N1 1.3261(18), C1-N2 1.3407(17), N2-N31.3773(15),N3-C2 1.2743(17), C2-C3 1.4612(18), C5-F1 1.3478(18), C6-F2 1.3511(16); S1-C1-N1 122.76(11), S1-C1-N2 120.24(10), N1-C1-N2 116.99(13), N2-N3-C2 115.55(11), N3-C2-C3 120.89(13).

**Figure 5:**
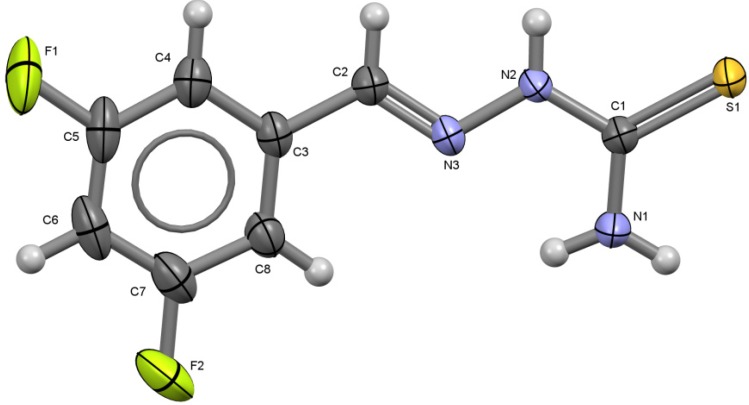
ORTEP drawing of 3,5-C_6_H_3_F_2_-CH=N-NH-C(S)NH_2_ (**5**). Thermal ellipsoids are shown at 50% of probability. Selected bond lengths (Å) and angles (°): S1-C1 1.6890(14), C1-N1 1.3164(19), C1-N2 1.3439(19), N2-N3 1.3699(16), N3-C2 1.2769(19), C2-C3 1.464(2), C5-F1 1.357(2), C7-F2 1.357(2); S1-C1-N1 123.19(12), S1-C1-N2 119.64(11), N1-C1-N2 117.17(13), N2-N3-C2 116.29(12), N3-C2-C3 119.68(14).

**Figure 6:**
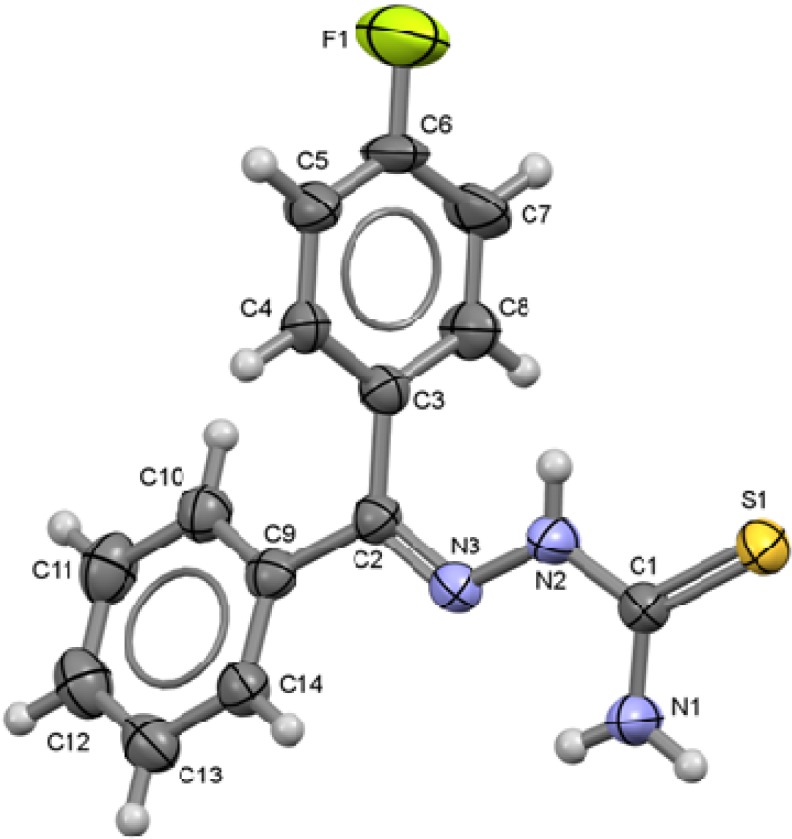
ORTEP drawing of 4-C_6_H_4_F-C(C_6_H_5_)=N-NH-C(S)NH_2_ (**6**). Thermal ellipsoids are shown at 50% of probability. Selected bond lengths (Å) and angles (°): S1-C1 1.686(5), C1-N1 1.316(5), C1-N2 1.347(6), N2-N3 1.374(5), N3-C2 1.278(6), C2-C3 1.495(5), C2-C9 1.488(6), C6-F1 1.346(6); S1-C1-N1 123.8(3), S1-C1-N2 119.3(3), N1-C1-N2 116.9(4), N2-N3-C2 118.1(3), N3-C2-C3 123.8(4), N3-C2-C9 116.5(4).

The IR spectra of compounds **1** to **6** show the expected signals from the polyfluoroaldehydes and the thiosemicarbazone groups, respectively [17]. The ^1^H-NMR spectra of compounds **1** to **6** exhibit the expected aromatic signals at *ca.* 8–11 ppm. In the thiosemicarbazones’ spectra there is a broad singlet at low field attributed to the NH- group and two broad singlets between 7 and 8 ppm assigned to each hydrogen atom in the -NH_2_ group, which show different magnetic behavior due to the double bond character in the C-N bond restricting its free rotation. Compounds **1**–**5** show a singlet at *ca.* 8 ppm due to the CH = N group [18].

In the solid state the thiosemicarbazone moieties in compounds **1**–**6** show an *E* configuration with the sulfur atom *trans* to the iminic nitrogen N3. All synthesized thiosemicarbazones exhibit the thione tautomeric form both in solution and in the solid state. This form is favored by the formation of the intramolecular N(1)-H---N(3) hydrogen bridge. Intramolecular and intermolecular hydrogen bonding occurs in all six molecules [19].

**Figure 7:**
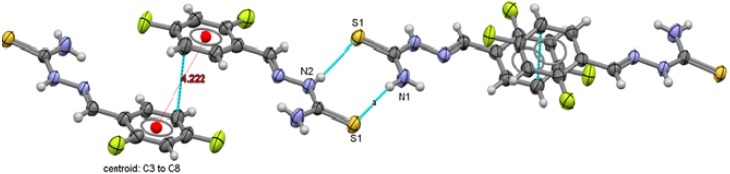
Packing arrangement of 2,4-C_6_H_3_F_2_-CH=N-NH-C(S)NH_2_ (**1**) showing the π interactions between thiophenolate rings.

The crystalline structures seem to be stabilized by the effect of N-H---S hydrogen bridges. As expected the relative position of the fluorine substituents on the aromatic ring of compounds **1** to **6** has a noticeable influence, not only on the chemical behavior of these compounds, but also on the different crystal packing arrangements, as shown in Figure 7 to Figure 12. The C2-N3 bond distances exhibit the expected values for a double bond. C1-S1 bond distances (average: 1.68 Å) are in between those of a C-S single bond (1.82 Å) and a C=S double bond (1.56 Å) thus reflecting a partial double bond character [20]. Both C1-N1 and C1-N2 bond lengths are shorter than the distance expected for a single C-N bond. N2-N3 bond length is also shorter than expected for a single N-N bond and therefore a delocalization seems to be present on this fragment.

**Figure 8:**
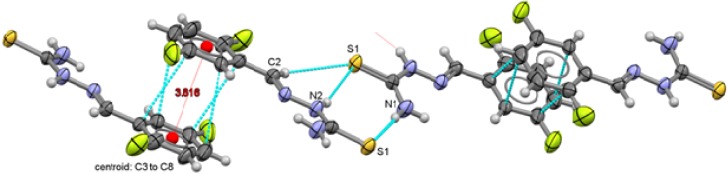
Packing arrangement of 2,5-C_6_H_3_F_2_-CH=N-NH-C(S)NH_2_ (**2**) showing the π interactions between thiophenolate rings.

**Figure 9:**
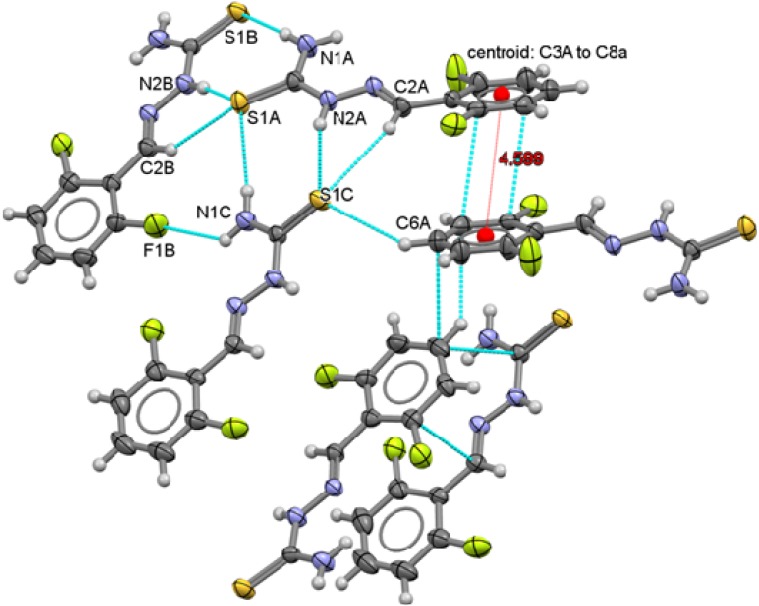
Packing arrangement of 2,6-C_6_H_3_F_2_-CH=N-NH-C(S)NH_2_ (**3**) showing the π interactions between thiophenolate rings.

**Figure 10:**
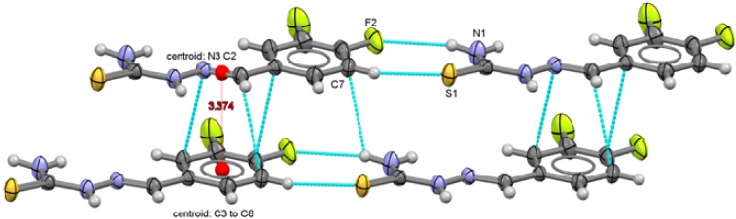
Packing arrangement of 3,4-C_6_H_3_F_2_-CH=N-NH-C(S)NH_2_ (**4**) showing the π interactions between thiophenolate rings.

**Figure 11:**
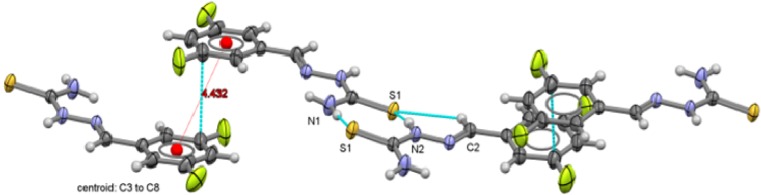
Packing arrangement of 3,5-C_6_H_3_F_2_-CH=N-NH-C(S)NH_2_ (**5**) showing the π interactions between thiophenolate rings.

**Figure 12:**
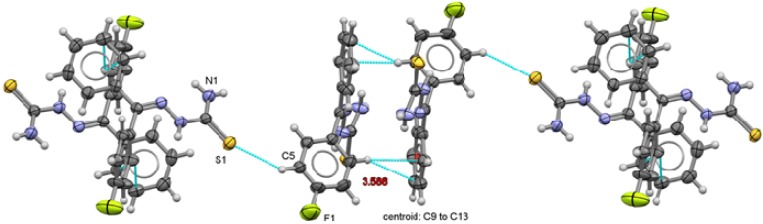
Packing arrangement of 4-C_6_H_4_F-C(C_6_H_5_)=N-NH-C(S)NH_2_ (**6**) showing the π interactions between thiophenolate rings.

Two types of intermolecular interactions are observed in the supramolecular structures of compounds **1** to **6**, there are π interactions between aromatic rings from neighbor molecules and also, there are intermolecular hydrogen-bond interactions with a 2.39 Å minimum distance and 2.89 Å as a maximum distance interaction.

## 3. Experimental

### 3.1. General

All reactions were carried out under dry, oxygen-free N_2_ atmospheres, using Schlenk techniques. Solvents were dried and degassed prior to use, using standard techniques [21]. Microanalyses were performed using a Fisons EA1108 instrument. FT-IR spectra were recorded over the 4000–200 cm^−1^range on a Nicolet Impact 4100 FT-IR spectrometer using KBr pellets. Data are expressed in wavenumbers (cm^−1^). ^1^H-, ^13^C- and ^19^F-NMR spectra were measured with a Varian Unity INOVA 300 MHz spectrometer operating at 299.7, 282, and 75 MHz, respectively and were collected at 25 °C, using D_6_-acetone for the deuterium lock. Chemical shifts are relative to TMS (δ = 0 (^1^H and ^13^C)) and CFCl_3_ (δ = 0 (^19^F)). Compounds were dissolved in deuterated acetone. Electronic Impact mass spectra were obtained on a JEOL JMS-SX102A instrument. All reagents were commercially available and were used as received.

### 3.2. General Procedure for the Synthesis of Fluorobenzylidene)hydrazine-1-carbothioamides ***1***–***6***

The corresponding Difluorobenzaldehyde (*ca* 5 mmol) was dissolved in ethanol (10 mL) at room temperature and added dropwise to a stirred solution of hydrazinecarbothioamide (*ca* 5 mmol), in a 1:1 mixture of ethanol/water (30 mL) containing 0.2 mL of acetic acid (Scheme 1). The reaction mixture was refluxed for 24 h. and then the solvent was reduced under vacuum to one third of the original value, from which the product precipitated as white crystalline solid. Recrystallization from ethanol/water (1:1) yielded the corresponding target product **1**–**5**.

*2-(2,4-Difluorobenzylidene)hydrazine-1-carbothioamide* (**1)**. From 2,4-difluorobenzaldehyde (0.8268 g, 3.84 mmol, 82%); colorless crystals, mp. 186–187 °C. ^1^H-NMR ((CD_3_)_2_CO) δ 7.95 (bs, 1H, N**H**_2_), δ 7.60 (bs, 1H, N**H**_2_), δ 10.65 (bs, 1H, N**H**), δ 8.37 (s, 1H, C2**H**), δ 8.22 (m, 1H, C5**H**), δ 7.09 (m, 2H, C7**H** and C8**H**); ^13^C-NMR ((CD_3_)_2_CO): δ (ppm) 179.77 (s, C-1), 163.78 (dd, C-6, ^1^*J*_CF_ = 251.1 Hz, ^3^*J*_CF_ = 12.4 Hz), 161.56 (dd, C-4, ^1^*J*_CF_ = 253.0 Hz, ^3^*J*_CF_ = 12.2 Hz), 134.32 (dd, C-2, ^3^*J*_CF_ = 4.7 Hz), 128.21 (dd, C-8, ^3^*J*_CF_ = 9.9 Hz, ^3^*J*_CF_ = 4.3 Hz), 118.77 (dd, C-3, ^2^*J*_CF_ = 10.2 Hz, ^4^*J*_CF_ = 3.8 Hz), 112.17 (dd, C-7, ^2^*J*_CF_ = 22.0, Hz, ^4^*J*_CF_ = 3.5 Hz), 103.93 (t, C-5, ^2^*J*_CF_ = 25.7 Hz). ^19^F{^1^H}-NMR ((CD_3_)_2_CO) δ -107.78 (m, 1F, C2**F**), δ −117.72 (m, 1F, C4**F**). Analysis results for C_8_H_7_F_2_N_3_S: Found: C, 45.09; H, 3.02; N, 19.05; S, 15.28. Calculated: C, 44.66; H, 3.28; N, 19.52; S, 14.90. IR (KBr, ν cm^−1^): C ν_as_(NH_2_) 3400, ν_s_(NH_2_) 3240, ν(NH) 3154, ν (C = N)/ν(C = C) 1600, ν(C-F) 1499, 1142, ν(C = S) 851. MS-IE: [M]^+^*m/z* 215 (100%).

*2-(2,5-Difluorobenzylidene)hydrazine-1-carbothioamide* (**2**). From 2,5-difluorobenzaldehyde (0.8050 g, 3.74 mmol, 81%); colorless crystals, mp. 187–188 °C. ^1^H-NMR ((CD_3_)_2_CO) δ 8.12 (bs, 1H, N**H**_2_), δ 7.63 (bs, 1H, N**H**_2_), δ 10.67 (bs, 1H, N**H**), δ 8.37 (s, 1H, C2**H**), δ 7.24 (m, 2H, C5**H** and C6**H**), δ 7.96 (m, 1H, C8**H**); ^13^C-NMR ((CD_3_)_2_CO): 180.91 (s, C-1), 159.90 (dd, C-7, ^1^*J*_CF_ = 240.53 Hz, ^4^*J*_CF_ = 2.1 Hz), 158.50 (dd, C-4, ^1^*J*_CF_ = 246.5 Hz, ^4^*J*_CF_ = 2.4 Hz), 134.73 (dd, C-2, ^3^*J*_CF_ = 4.9 Hz), 124.56 (dd, C-3, ^2^*J*_CF_ = 12.4 Hz, ^3^*J*_CF_ = 8.6 Hz), 118.98 (dd, C-8, ^2^*J*_CF_ = 25.3 Hz, ^3^*J*_CF_ = 8.9 Hz), 118.32 (dd, C-6, ^2^*J*_CF_ = 24.3 Hz, ^3^*J*_CF_ = 8.8 Hz), 113.06 (dd, C-5, ^2^*J*_CF_ = 26.0, Hz, ^3^*J*_CF_ = 3.3 Hz). ^19^F{^1^H}NMR ((CD_3_)_2_CO) δ −119.77 (m, 1F, C2**F**), δ −128.40 (m, 1F, C5**F**). Analysis results for C_8_H_7_F_2_N_3_S: Found: C, 44.35; H, 3.14; N, 19.53; S, 15.7. Calculated: C, 44.66; H, 3.28; N, 19.52; S, 14.90. IR (KBr, cm^−1^): ν_as_(NH_2_) 3405, ν_s_(NH_2_) 3243, ν(NH) 3160, ν(C = N)/ν(C = C) 1601, ν(C-F) 1488, 1115, ν(C = S) 820. MS-IE: [M]^+^*m/z* 215 (10%).

*2-(2,6-Difluorobenzylidene)hydrazine-1-carbothioamide* **(3**). From 2,6-difluorobenzaldehyde (0.9200 g, 4.28 mmol, 92%); colorless crystals, mp. 195–196 °C. ^1^H-NMR ((CD_3_)_2_CO) δ 7.60 (bs, 1H, N**H**_2_), δ 7.50 (m, 1H, N**H**_2_), δ 10.66 (bs, 1H, N**H**), δ 8.32 (s, 1H, C2**H**), δ 7.11 (m, 2H, C5**H** and C7**H**), δ 7.50 (m, H, C6**H**); ^13^C-NMR ((CD_3_)_2_CO): δ (ppm) 180.81 (s, C-1), 162.02 (dd, C-4, ^1^*J*_CF_ = 255.2 Hz, ^3^*J*_CF_ = 6.5 Hz), 162.00 (dd, C-8, ^1^*J*_CF_ = 255.2 Hz, ^3^*J*_CF_ = 6.5 Hz), 133.44 (s, C-2), 132.46 (t, C-6, ^3^*J*_CF_ = 10.8 Hz), 113.01 (m, C-5, C-7), 112.56 (t, C-3, ^2^*J*_CF_ = 13.5 Hz). ^19^F{^1^H}-NMR ((CD_3_)_2_CO) δ −113.02 (m, 1F, C2**F**), δ −113.02 (m, 1F, C6**F**). Analysis results for C_8_H_7_F_2_N_3_S: Found: C, 45.01; H, 3.34; N, 19.56; S, 15.75. Calculated: C, 44.66; H, 3.28; N, 19.52; S, 14.90. IR (KBr, cm^−1^): ν_as_(NH_2_) 3430, 3410, ν_s_(NH_2_) 3264, ν(NH) 3158, ν(C = N)/ν(C = C) 1601, ν(C-F) 1459, 1150, ν(C = S) 876. MS-EI: [M]^+^*m/z* 215 (70%).

*2-(3,4-Difluorobenzylidene)hydrazine-1-carbothioamide* (**4)**. From 3,4-difluorobenzaldehyde (0.9500 g, 4.42 mmol, 95%); colorless crystals, mp. 196–197 °C. ^1^H-NMR ((CD_3_)_2_CO) δ 8.05 (bs, 1H, N**H**_2_), δ 7.58 (m, 1H, N**H**_2_), δ 10.56 (bs, 1H, N**H**), δ 8.15 (s, 1H, C2**H**), δ 7.58 (m, H, C4**H**), δ 7.38 (m, 1H, C7**H**), δ 7.94 (m, 1H, C8**H**); ^13^C-NMR ((CD_3_)_2_CO): δ (ppm) 180.72 (s, C-1), 152.00 (dd, C-6, ^1^*J*_CF_ = 250.0 Hz, ^2^*J*_CF_ = 13.1 Hz), 151.47 (dd, C-5, ^1^*J*_CF_ = 246.3 Hz, ^2^*J*_CF_ = 13.1 Hz), 140.99 (t, C-2, ^4^*J*_CF_ = 2.4 Hz), 133.13 (dd, C-3, ^3^*J*_CF_ = 6.5 Hz, ^4^*J*_CF_ = 3.8 Hz), 125.82 (dd, C-8, ^3^*J*_CF_ = 6.7 Hz, ^4^*J*_CF_ = 3.4 Hz), 118.50 (d, C-4, ^2^*J*_CF_ = 17.9 Hz), 115.82 (d, C-7, ^2^*J*_CF_ = 18.7 Hz). ^19^F{^1^H}-NMR ((CD_3_)_2_CO) δ -136.97 (m, 1F, C3**F**), δ −138.82 (m, 1F, C4**F**). Analysis results for C_8_H_7_F_2_N_3_S: Found: C, 44.64; H, 3.21; N, 19.32; S, 14.82. Calculated: C, 44.66; H, 3.28; N, 19.52; S, 14.90. IR (KBr, ν cm^−1^): ν_as_(NH_2_) 3426, ν_s_(NH_2_) 3273, ν(NH) 3167, ν(C = N)/ν(C = C) 1609, ν(C-F) 1510, 1158, ν(C = S) 828. MS-EI: [M]^+^*m/z* 215 (75%).

*2-(3,5-Difluorobenzylidene)hydrazine-1-carbothioamide* (**5)**. From 3,5-difluorobenzaldehyde (0.8620 g, 4.01 mmol, 86%); colorless crystals, mp. 196–197 °C. ^1^H-NMR ((CD_3_)_2_CO) δ 8.13 (bs, 1H, N**H**_2_), δ 7.67 (m, 1H, N**H**_2_), δ 10.68 (bs, 1H, N**H**), δ 8.16 (s, 1H, C2**H**), δ 7.51 (m, H, C4**H**), δ 7.05 (tt, 1H, C6**H**, ^3^*J*_HF_ = 9.1 Hz, ^4^*J*_HF_ = 2.4 Hz), δ 7.51 (m, 1H, C8**H**); ^13^C-NMR ((CD_3_)_2_CO): δ (ppm) 179.92 (s, C-1), 163.27 (dd, C-5, ^1^*J*_CF_ = 246.6 Hz, ^3^*J*_CF_ = 12.9 Hz), 163.14 (dd, C-7, ^1^*J*_CF_ = 246.6 Hz, ^3^*J*_CF_ = 12.9 Hz), 139.74 (t, C-2, ^4^*J*_CF_ = 3.6 Hz), 138.31 (t, C-3, ^3^*J*_CF_ = 9.9 Hz), 109.88 (dd, C-4, C-8, ^2^*J*_CF_ = 19.2 Hz, ^4^*J*_CF_ = 7.3 Hz), 104.60 (t, C-6, ^2^*J*_CF_ = 26.2 Hz). ^19^F{^1^H}-NMR ((CD_3_)_2_CO) δ -110.40 (m, 1F, C3**F**), δ −110.40 (m, 1F, C5**F**). Mp. 199–200 °C. Analysis results for C_8_H_7_F_2_N_3_S: Found: C, 44.74; H, 3.18; N, 19.79; S, 15.06. Calculated: C, 44.66; H, 3.28; N, 19.52; S, 14.90. IR (KBr, ν cm^−1^): ν_as_(NH_2_) 3395, ν_s_(NH_2_) 3238, ν(NH) 3158, ν(C = N)/ν(C = C) 1605, ν(C-F) 1467, 1124, ν(C = S) 855. MS-EI: [M]^+^*m/z* 215 (20%).

*2-(4-Fluorophenyl)(phenyl)methylene)benzylidene)hydrazine-1-carbothioamide* (**6)**. From 4-fluoro-phenyl-(phenyl)methanone (0.8648 g, 4.02 mmol, 86%); colorless crystals, mp 198–199 °C. ^1^H-NMR ((CD_3_)_2_CO) δ 8.12 (bs, 1H, N**H**_2_), δ 7.76 (m, 1H, N**H**_2_), δ 8.52 (bs, 1H, N**H**), δ 7.70 (m, H, C4**H**), δ 7.15 (tt, 2H, C5**H**, ^3^J_HF_ = 9.9 Hz, ^3^J_HH_ = 8.8 Hz and , C7**H**,^3^J_HF_ = 9.9 Hz, ^3^J_HH_ = 8.8 Hz), δ 7.70 (m, 1H, C8**H**), δ 7.64 (m, 2H, C10**H** and C14**H**), δ 7.39 (m, 2H, C11**H** and C13**H**), δ 7.46 (m, 1H, C12**H**); ^13^C-NMR ((CD_3_)_2_CO): δ (ppm) 179.48 (s, C-1), 163.66 (d, C-6, ^1^*J*_CF_ = 248.58 Hz), 148.36 (C-2), 136.84 (s, C-9), 133.28 (d, C-3, ^4^*J*_CF_ = 3.1 Hz), 130.17 (s, C-12), 129.92 (s, C-10, C-14), 129.78 (d, C-4, C-8 ^3^*J*_CF_ = 8.5 Hz), 128.43 (s, C-11, C-13), 115.17 (d, C-5, C-7 ^2^*J*_CF_ = 21.9 Hz). ^19^F {^1^H}NMR ((CD_3_)_2_CO) δ -111.36 (m, 1F, C4**F**). mp. 205 °C. Analysis results for C_14_H_12_FN_3_S: Found: C, 61.53; H, 4.56; N, 15.47; S 11.71. Calculated: C, 61.52; H, 4.42; N, 15.37; S, 11.73. IR (KBr, ν cm^−1^): ν_as_(NH_2_) 3430, ν_s_(NH_2_) 3353, ν(NH) 3248, ν(C = N)/ν(C = C) 1595, ν(C-F) 1499, 1155, ν(C = S) 854. MS-EI: [M]^+^*m/z* 273 (14%).

### 3.3. X-ray Crystal Data for Compounds ***1*** to ***6***

Crystals **1**–**5** were studied using an Oxford Diffraction Gemini “A” diffractometer with a CCD area detector (*λ*_MoK__α_ = 0.71073 Å), while data for crystal **6** was collected on a Kappa CCD single crystal diffractometer with Mo-Kα radiation (λ = 0.71073 Å).

Crystal data: **1** C_8_H_7_F_2_N_3_S, M = 215.23, monoclinic, *P*2_1_/c, a = 12.0601(7), b = 8.1871(3), c = 10.6170(6) Å, *β* = 112.431(7)°, V = 968.98(9) Å^3^, Z = 4, D_c_ = 1.475 g.cm^−3^, *µ* = 0.326 mm^−1^, 2296 independent measured reflections, *F*^2^ refinement, *R*_1_ = 0.0342, *wR*2 = 0.0811, goodness-of-fit = 0.896, 139 parameters, 0 restrains; **2** C_8_H_7_F_2_N_3_S, M = 215.23, monoclinic, *P*2_1_/c, a = 10.3711(5), b = 8.3331(2), c = 12.0732(5) Å, *β* = 111.923(6)°, V = 967.95(7) Å^3^, Z = 4, D_c_ = 1.477 g.cm^−3^, *µ* = 0.327 mm^−1^, 2292 independent measured reflections, *F*^2^ refinement, *R*_1_ = 0.0377, *wR*2 = 0.1028, goodness-of-fit = 1.026, 127 parameters, 0 restrains. **3** C_8_H_7_F_2_N_3_S, M = 215.24, monoclinic, *P*2_1_/n, a = 7.9792(6), b = 15.1661(12), c = 23.6599(19) Å, *β* = 90.553(8)°, V = 2863.0(4) Å^3^, Z = 4, D_c_ = 1.498 g.cm^−3^, *µ* = 0.331 mm^−1^, 6848 independent measured reflections, *F*^2^ refinement, *R*_1_ = 0.0377, *wR*2 = 0.0934, goodness-of-fit = 1.025, 379 parameters, 0 restrains. **4** C_8_H_7_F_2_N_3_S, M = 215.23, triclinic, *P-1*, a = 4.5780(2), b = 9.1499(4), c = 11.7475(7) Å, *α =* 81.555(5), *β =* 84.955(4), *γ =* 77.972(4)°, V = 475.22(4) Å^3^, Z = 2, D_c_ = 1.504 g.cm^−3^, *µ* = 0.333 mm^−1^, 1880 independent measured reflections, *F*^2^ refinement, *R*_1_ = 0.0312, *wR*2 = 0.0898, goodness-of-fit = 1.105, 127 parameters, 0 restrains. **5** C_8_H_7_F_2_N_3_S, M = 215.23, monoclinic, *P*2_1_/c, a = 12.0810(8), b = 8.1670(3), c = 10.7870(5) Å, *β* = 113.635(7)°, V = 975.03(9) Å^3^, Z = 4, D_c_ = 1.466 g.cm^−3^, *µ* = 0.324 mm^−1^, 2309 independent measured reflections, *F*^2^ refinement, *R*_1_ = 0.0370, *wR*2 = 0.0990, goodness-of-fit = 0.958, 127 parameters, 0 restrains. **6** C_14_H_12_FN_3_S, M = 273.33, monoclinic, *C*2/c, a = 18.598(4) Å, b = 9.592(2) Å, c = 16.813(3)Å, *β* = 111.43(3)°, V = 2791.7(1)Å^3^, Z = 8, D_c_ = 1.301 g.cm^−3^, *µ* = 0.232 mm^−1^, 2466 independent measured reflections, *F*^2^ refinement, *R*_1_ = 0.0717, *wR*2 = 0.2334, goodness-of-fit = 1.040, 172 parameters, 0 restrains.

## 4. Conclusions

As expected, the reactions of hydrazinecarbothioamide with the fluorobenzaldehides R-CHO (R = 2,4-C_6_H_3_F_2_, R = 2,5-C_6_H_3_F_2_, R = 2,6-C_6_H_3_F_2_, R = 3,4-C_6_H_3_F_2_, R = 3,5-C_6_H_3_F_2_) or the fluoroacetone 4-C_6_H_4_F-C(Ph)O give rise to the six corresponding examples of fluorinated thiosemicarbazones, with very high yields. The x-ray diffraction molecular and crystal structures obtained for all six compounds, confirms that they all have an *E* configuration with the sulfur atom *trans* to the iminic nitrogen. The NH_2_ group at the carbothioamide moiety exhibit hydrogens with two different magnetic behavior due to the double bond character in the C-N bond restricting the free rotation of this bond. In addition, the crystal network structured by these compounds shows the presence of π interactions between some aromatic rings of neighboring molecules and several intermolecular interactions between atoms of hydrogen and sulfur or fluorine atoms.

## Supplementary Materials

Supplementary Materials include copies of IR, MS, ^1^H-, ^13^C- and ^19^F-NMR spectra for compounds **1**–**6**. These materials can be accessed at: http://www.mdpi.com/1420-3049/18/10/13111/s1.
