# Supplementary material for: Synthesis and Structural Characterization of Fluorinated Thiosemicarbazones
Source: Molecules. 2013 Oct 22;18(10):13111–23. doi: 10.3390/molecules181013111 (PMC6270490; doi:10.3390/molecules181013111)

Supplementary Materials

2-(2,4-Difluorobenzylidene)hydrazine-1-carbothioamide (**1**)

Figure S1. IR spectrum.

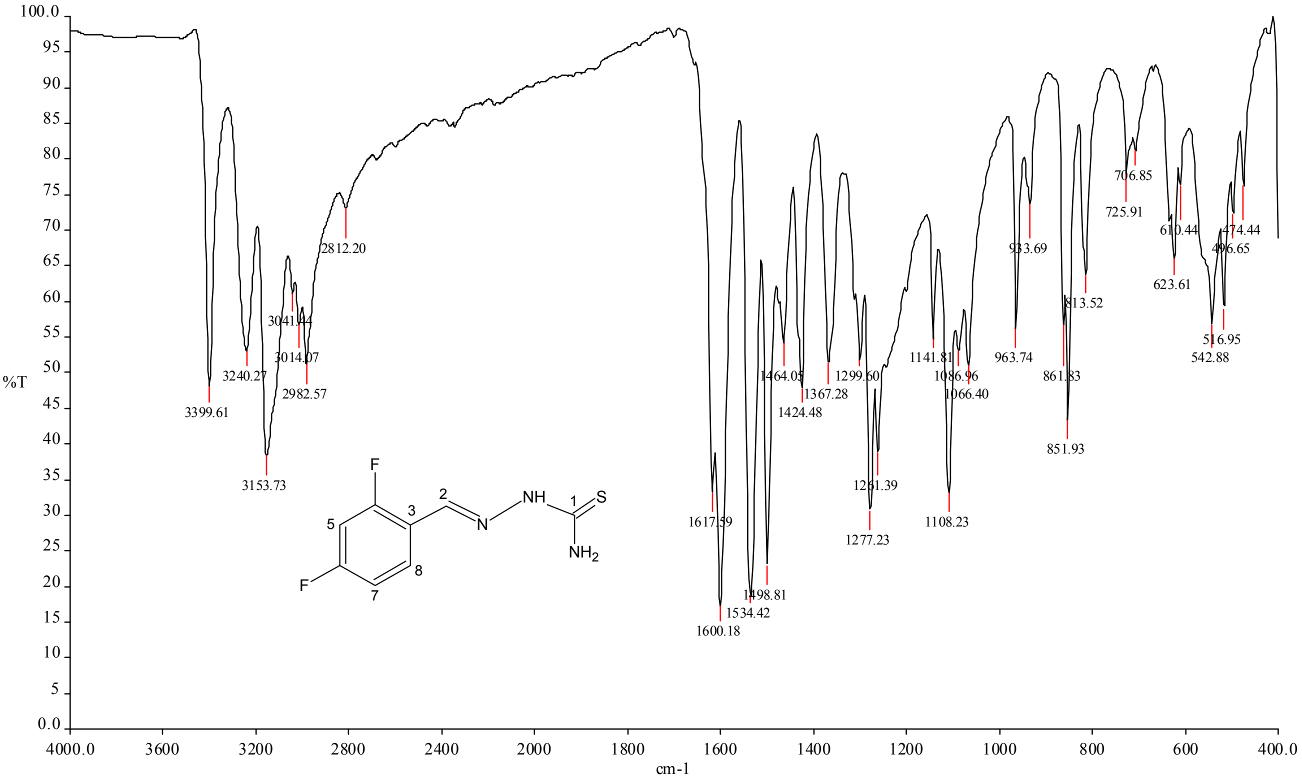

2-(2,4-Difluorobenzylidene)hydrazine-1-carbothioamide (**1**)

Figure S2. Mass spectrum.

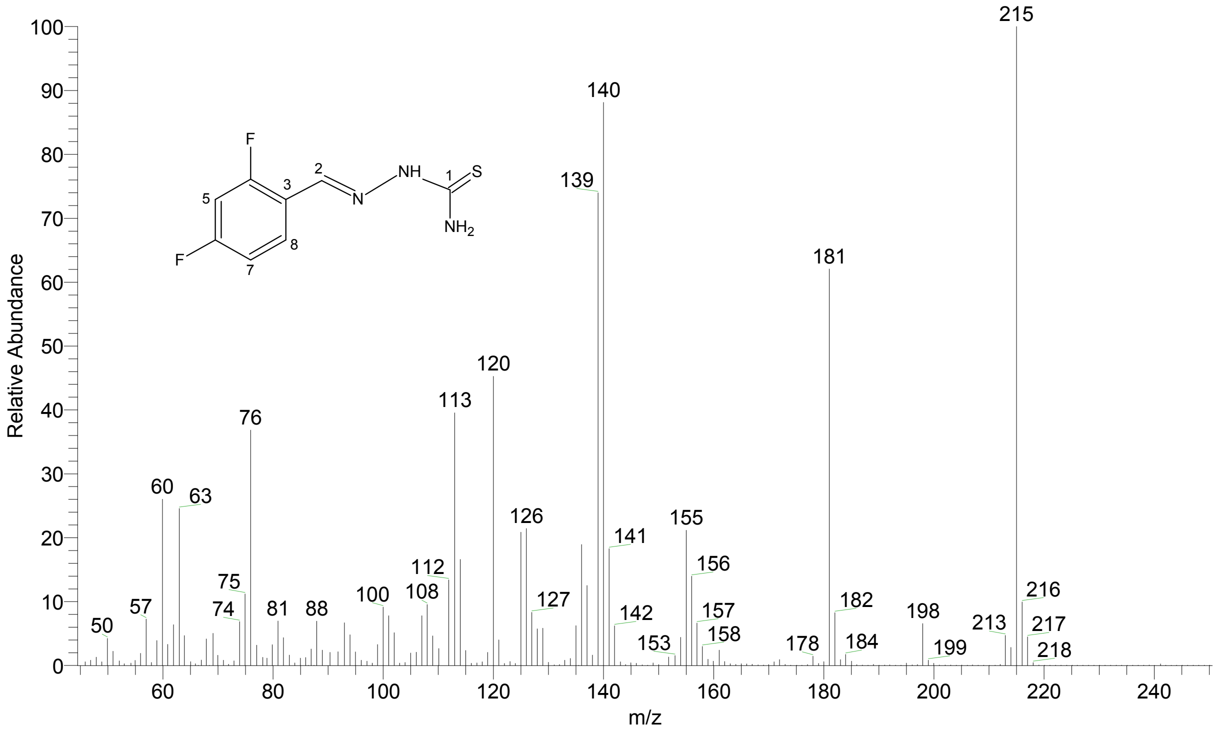

2-(2,4-Difluorobenzylidene)hydrazine-1-carbothioamide (**1**)

Figure S3. ^{1}H-NMR spectrum.

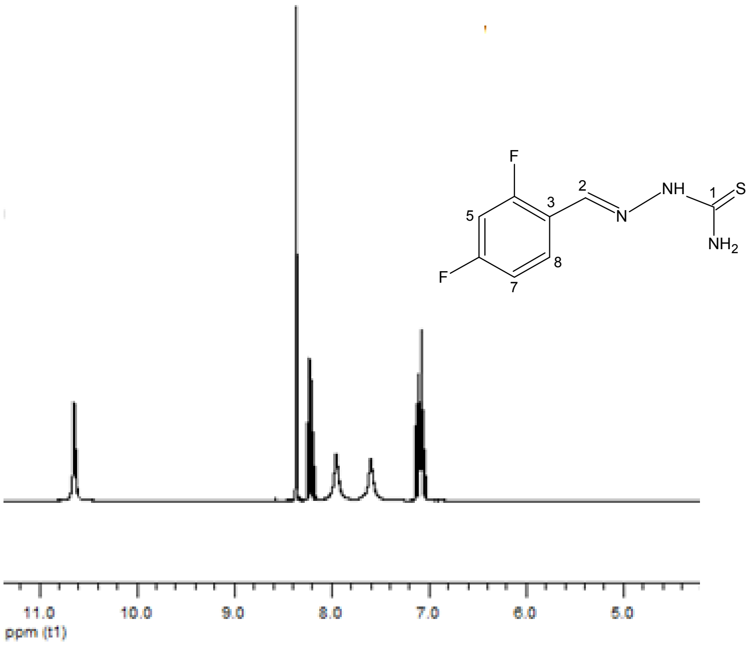

2-(2,4-Difluorobenzylidene)hydrazine-1-carbothioamide (**1**)

Figure S4. ^{13}C-NMR spectrum.

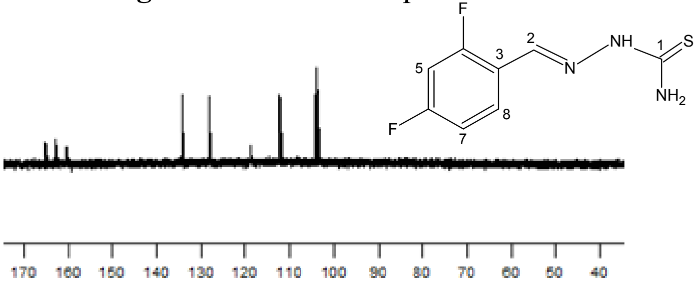

2-(2,4-Difluorobenzylidene)hydrazine-1-carbothioamide (**1**)

Figure S5. ^{19}F-NMR spectrum.

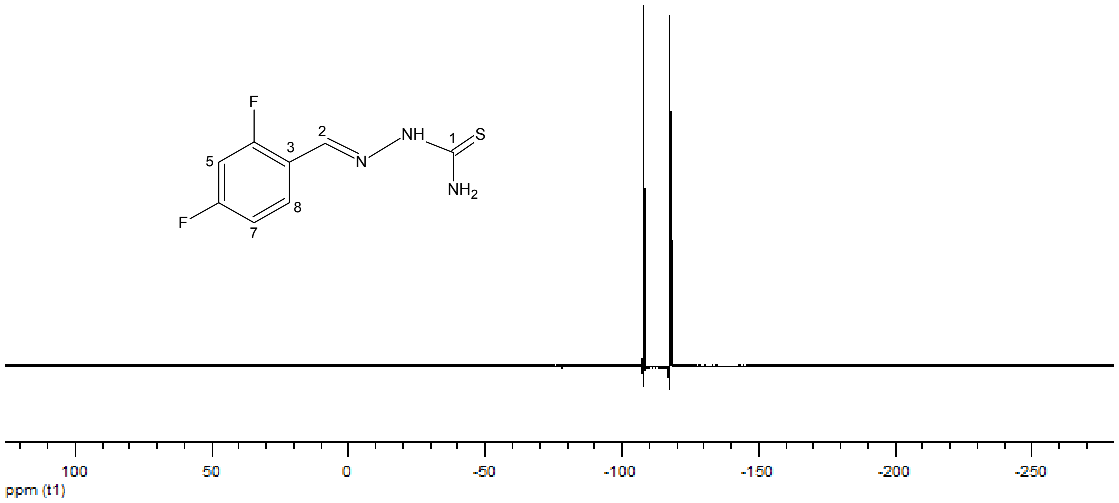

2-(2,5-Difluorobenzylidene)hydrazine-1-carbothioamide (**2**)

Figure S6. IR spectrum.

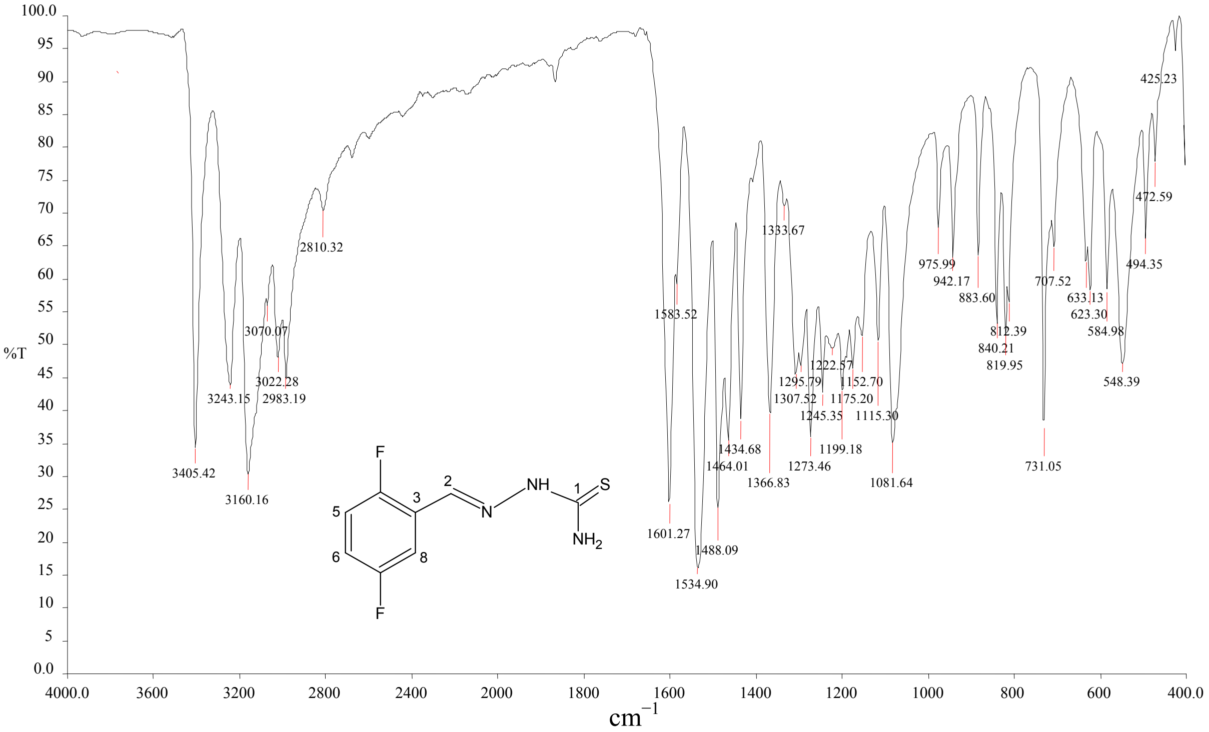

2-(2,5-Difluorobenzylidene)hydrazine-1-carbothioamide (**2**)

Figure S7. Mass spectrum.

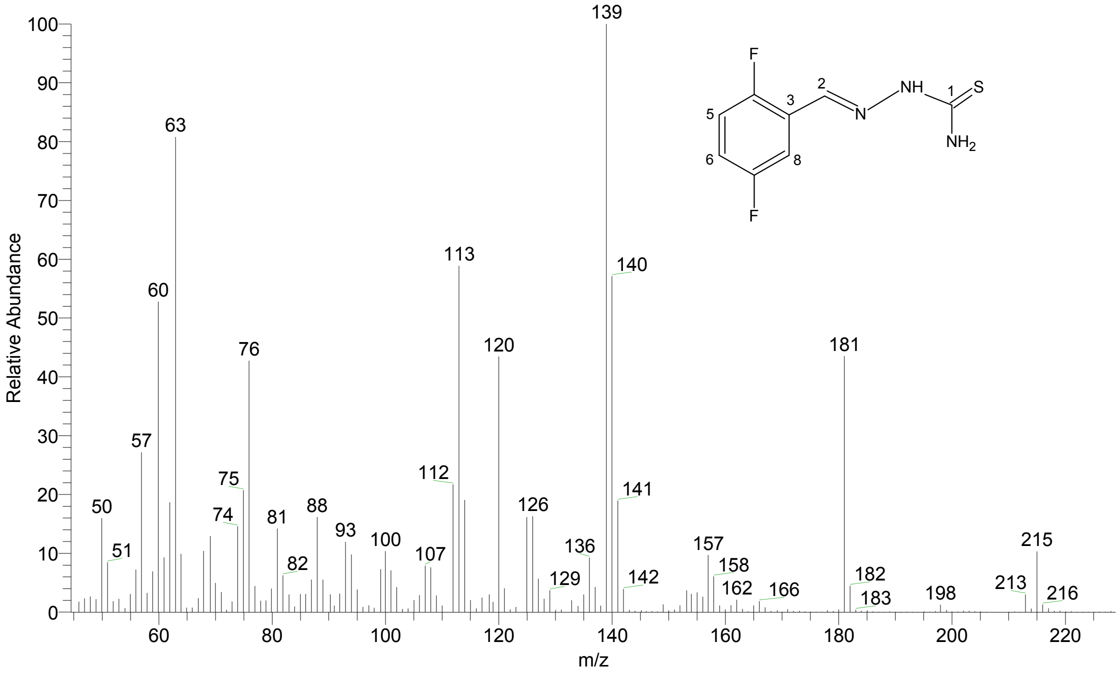

2-(2,5-Difluorobenzylidene)hydrazine-1-carbothioamide (**2**)

Figure S8. ^{1}H-NMR spectrum.

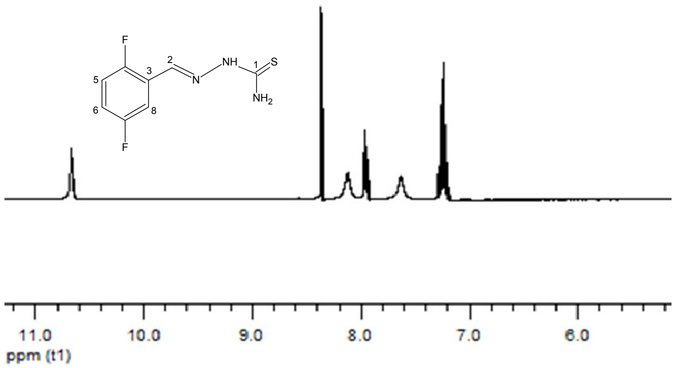

2-(2,5-Difluorobenzylidene)hydrazine-1-carbothioamide (**2**)

Figure S9. ^{13}C-NMR spectrum.

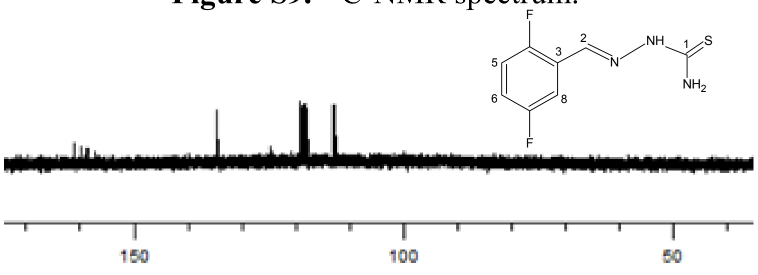

2-(2,5-Difluorobenzylidene)hydrazine-1-carbothioamide (**2**)

Figure S10. ^{19}F-NMR spectrum.

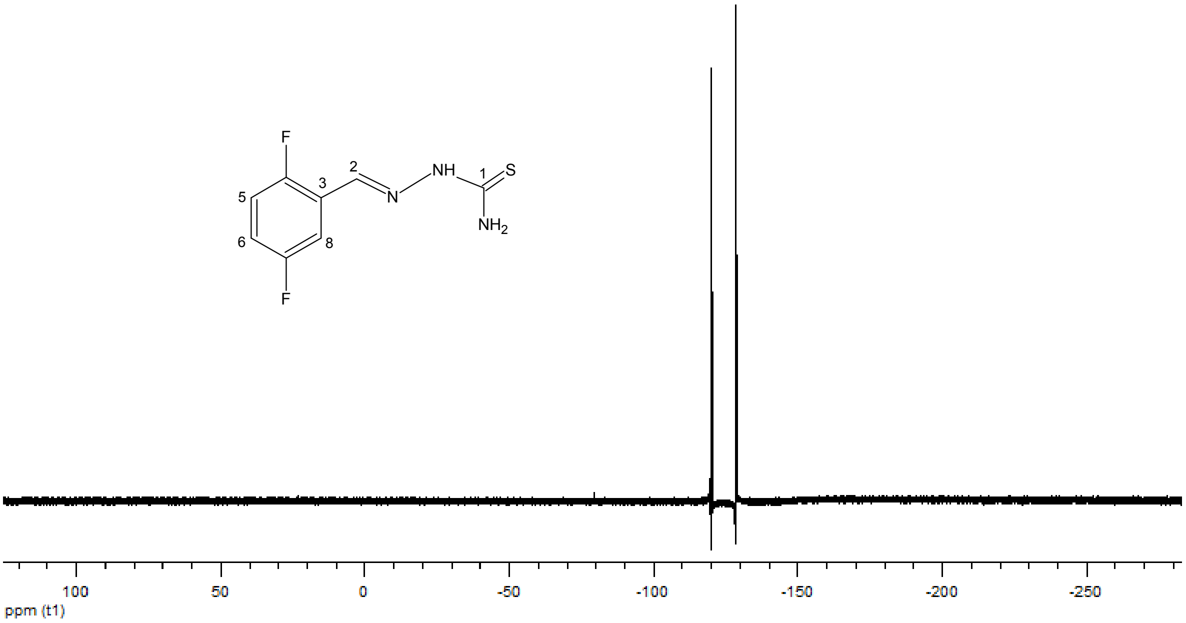

2-(2,6-Difluorobenzylidene)hydrazine-1-carbothioamide (**3**)

Figure S11. IR spectrum.

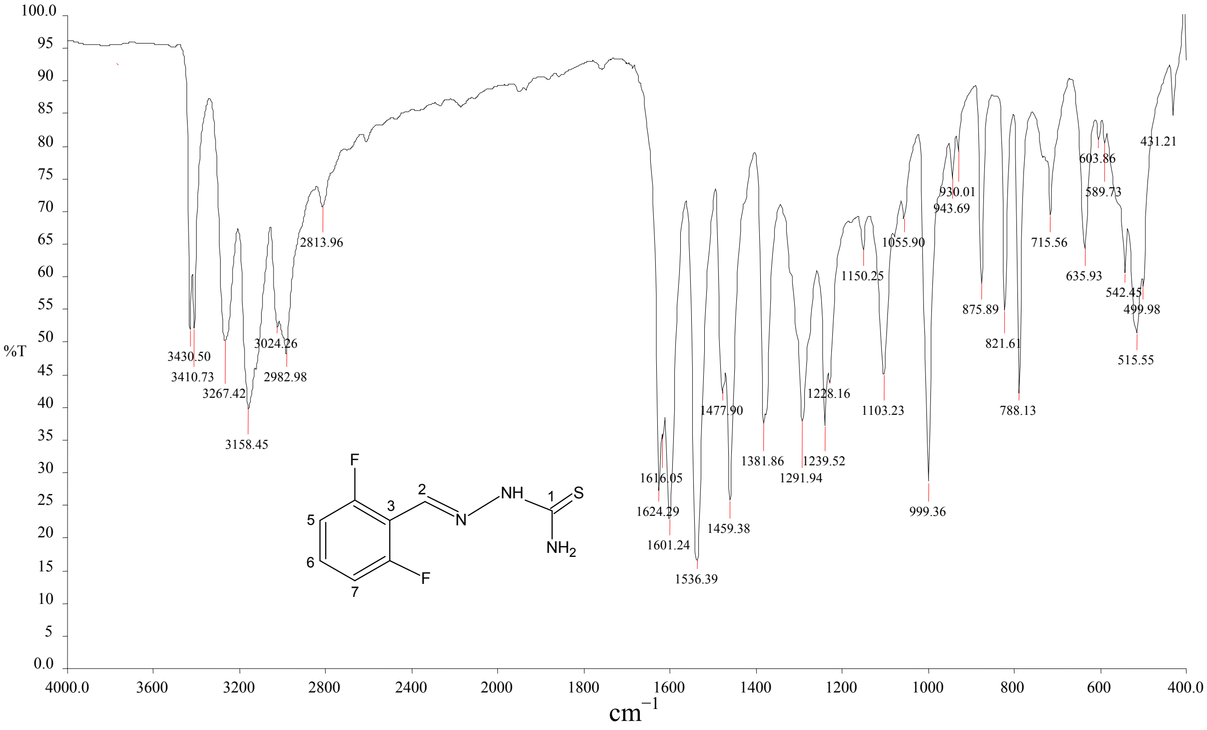

2-(2,6-Difluorobenzylidene)hydrazine-1-carbothioamide (**3**)

Figure S12. Mass spectrum.

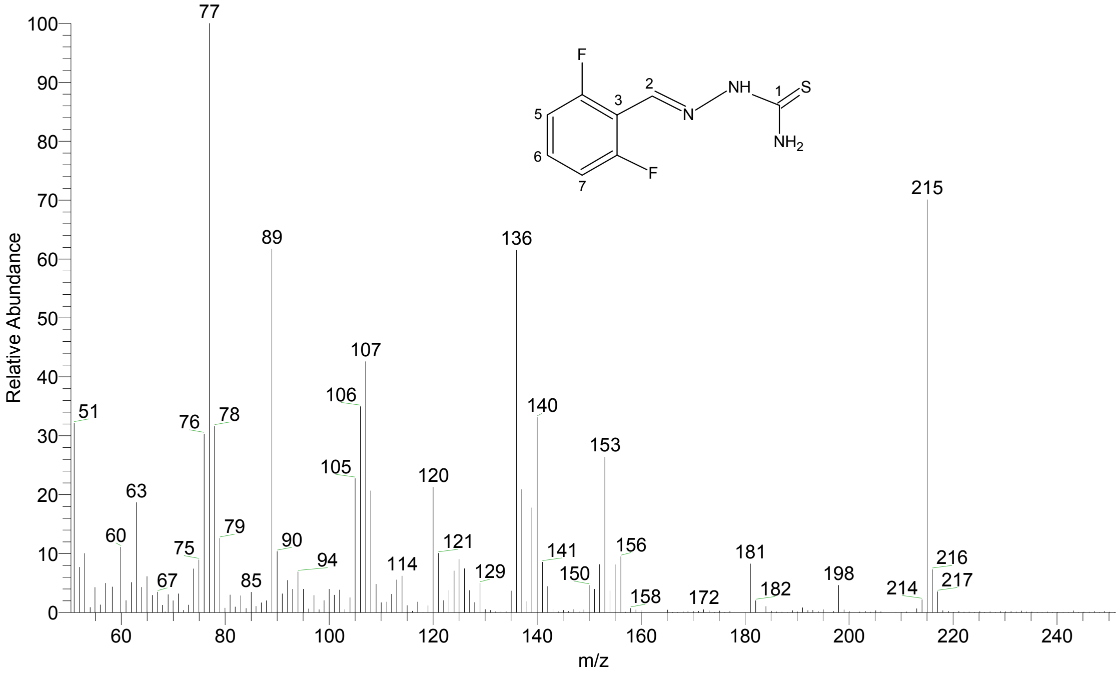

2-(2,6-Difluorobenzylidene)hydrazine-1-carbothioamide (**3**)

Figure S13. ^{1}H-NMR spectrum.

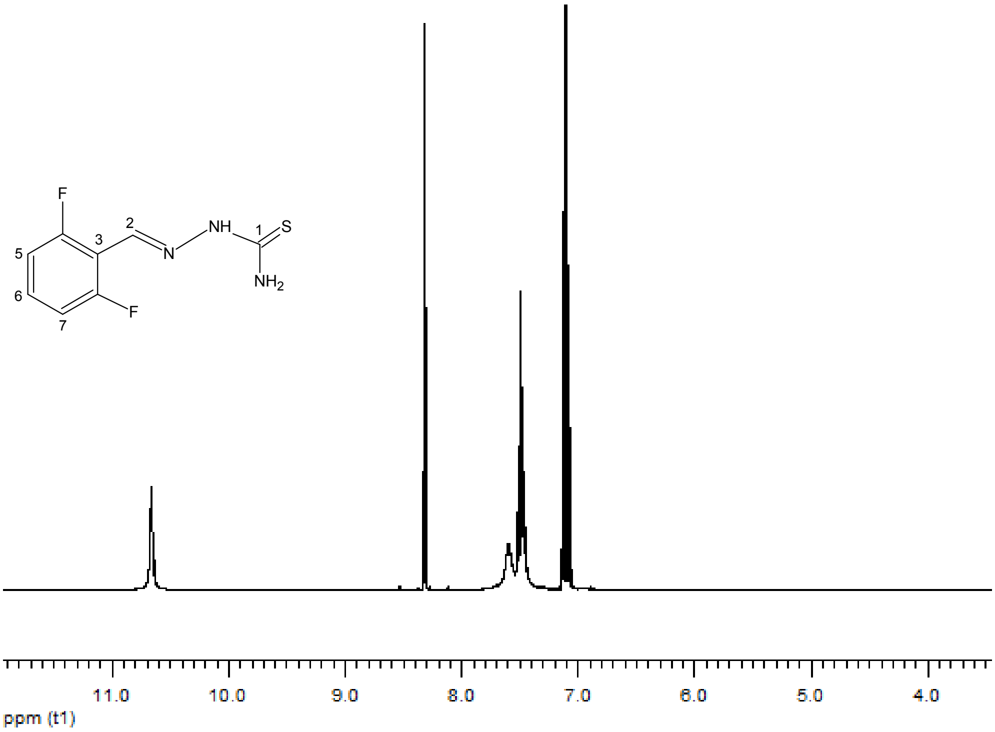

2-(2,6-Difluorobenzylidene)hydrazine-1-carbothioamide (**3**)

Figure S14. ^{13}C-NMR spectrum.

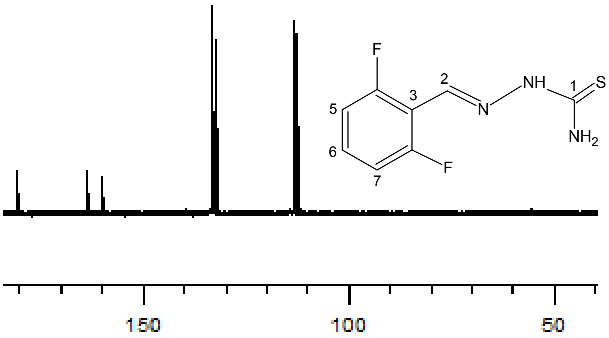

2-(2,6-Difluorobenzylidene)hydrazine-1-carbothioamide (**3**)

Figure S15. ^{19}F-NMR spectrum.

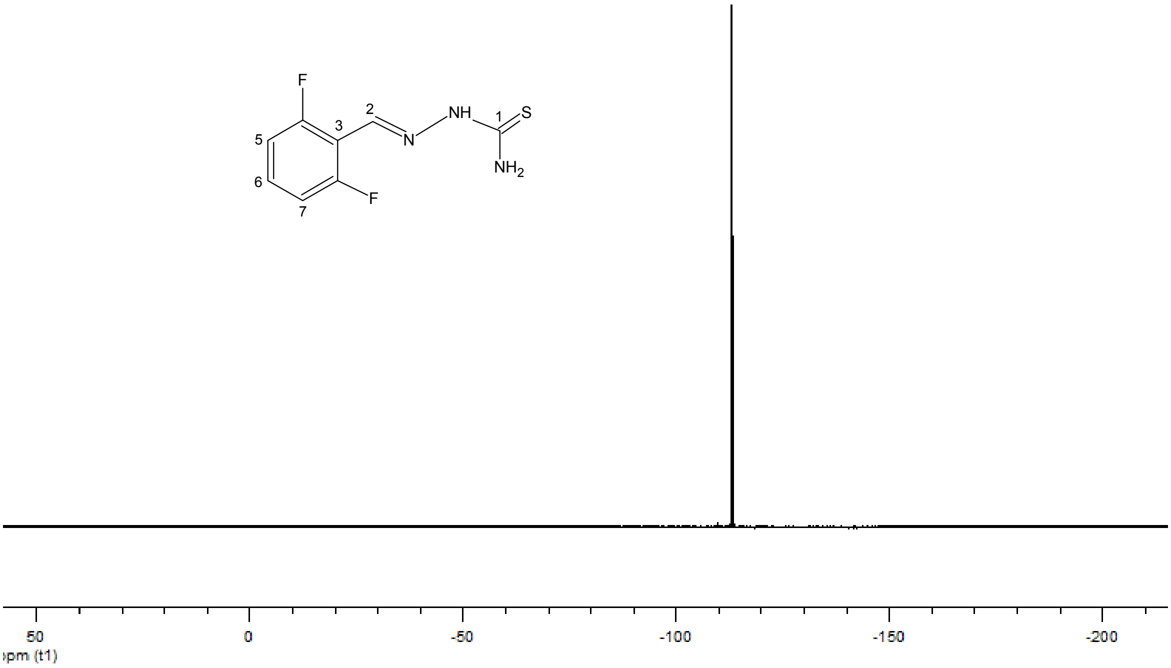

2-(3,4-Difluorobenzylidene)hydrazine-1-carbothioamide (**4**)

Figure S16. IR spectrum.

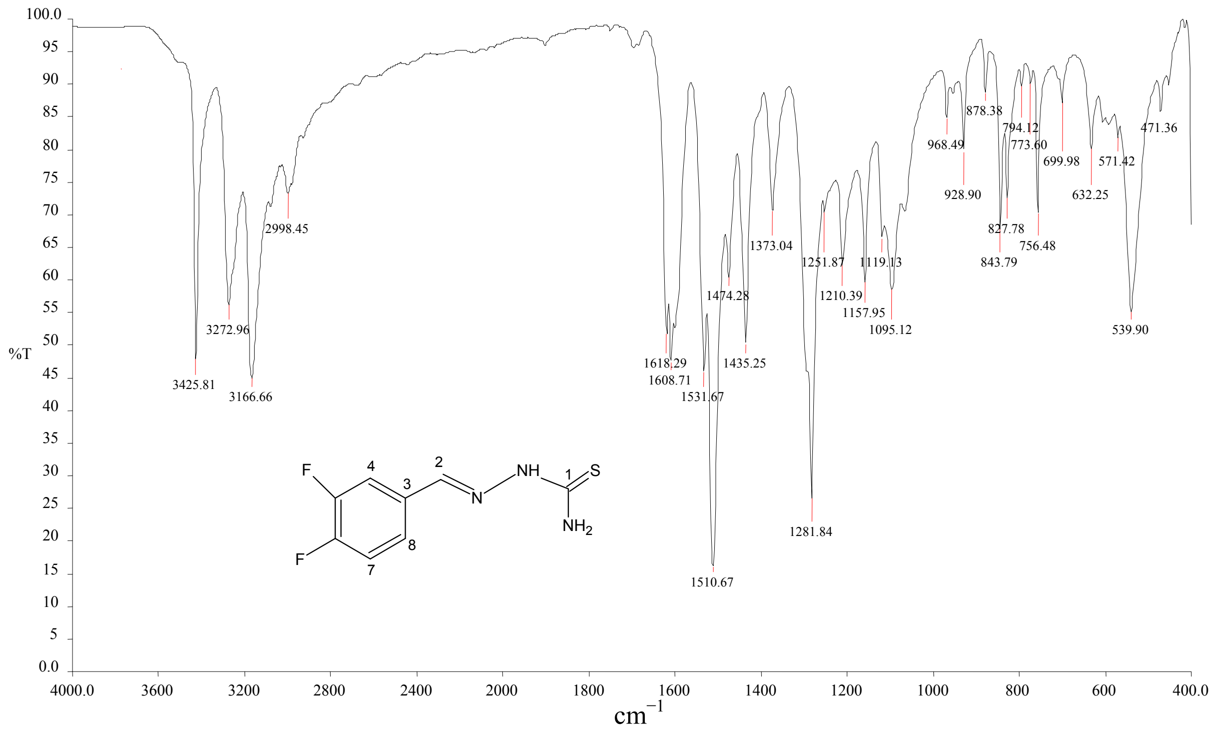

2-(3,4-Difluorobenzylidene)hydrazine-1-carbothioamide (**4**)

Figure S17. Massspectrum.

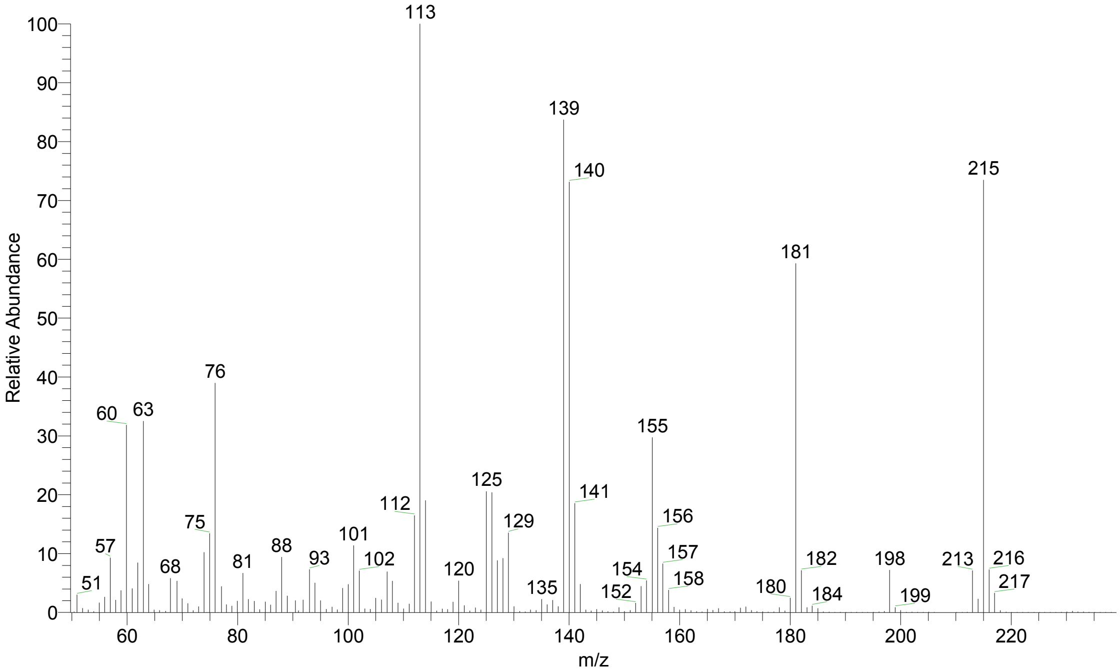

2-(3,4-Difluorobenzylidene)hydrazine-1-carbothioamide (**4**)

Figure S18. ^{1}H-NMR spectrum.

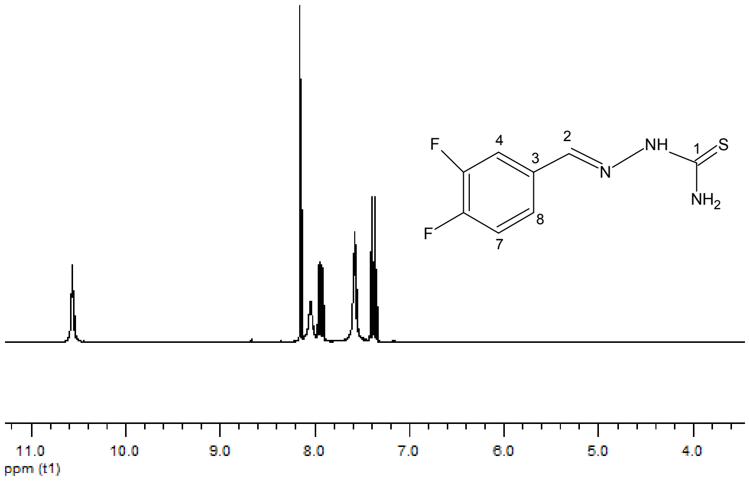

2-(3,4-Difluorobenzylidene)hydrazine-1-carbothioamide (**4**)

Figure S19. ^{13}C-NMR spectrum.

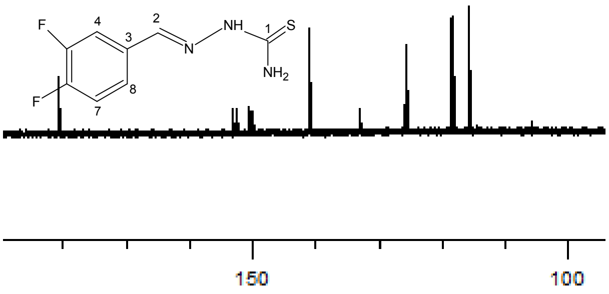

2-(3,4-Difluorobenzylidene)hydrazine-1-carbothioamide (**4**)

Figure S20. ^{19}F-NMR spectrum.

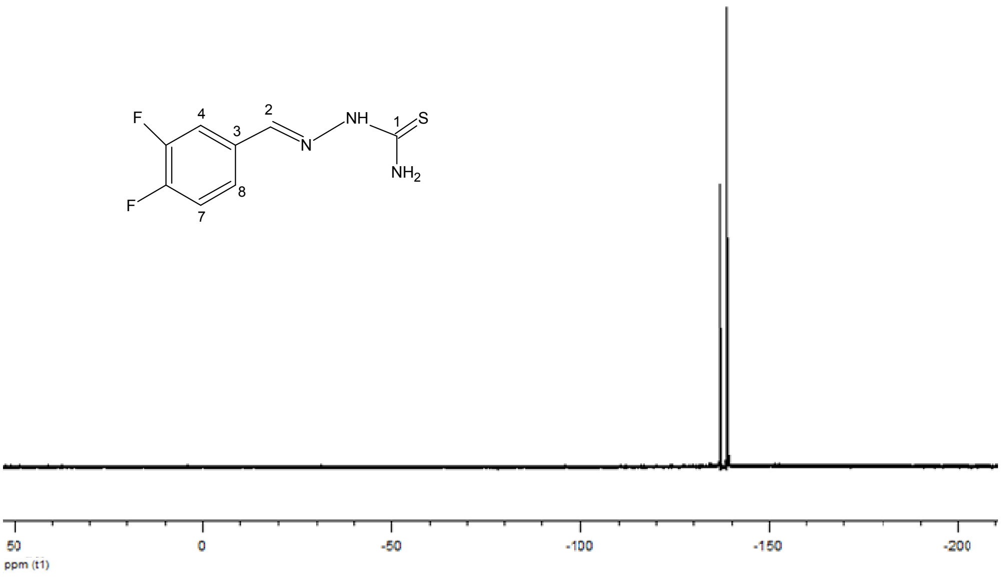

2-(3,5-Difluorobenzylidene)hydrazine-1-carbothioamide (**5**)

Figure S21. IR spectrum.

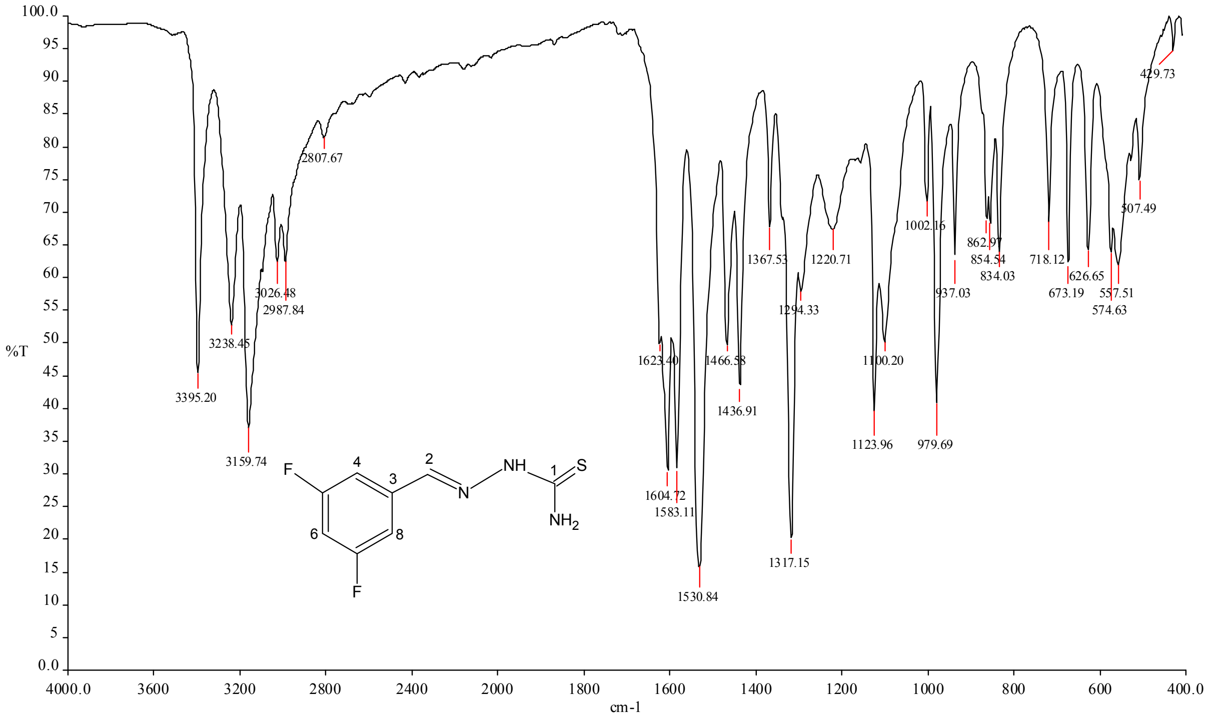

2-(3,5-Difluorobenzylidene)hydrazine-1-carbothioamide (**5**)

Figure 22. Mass spectrum.

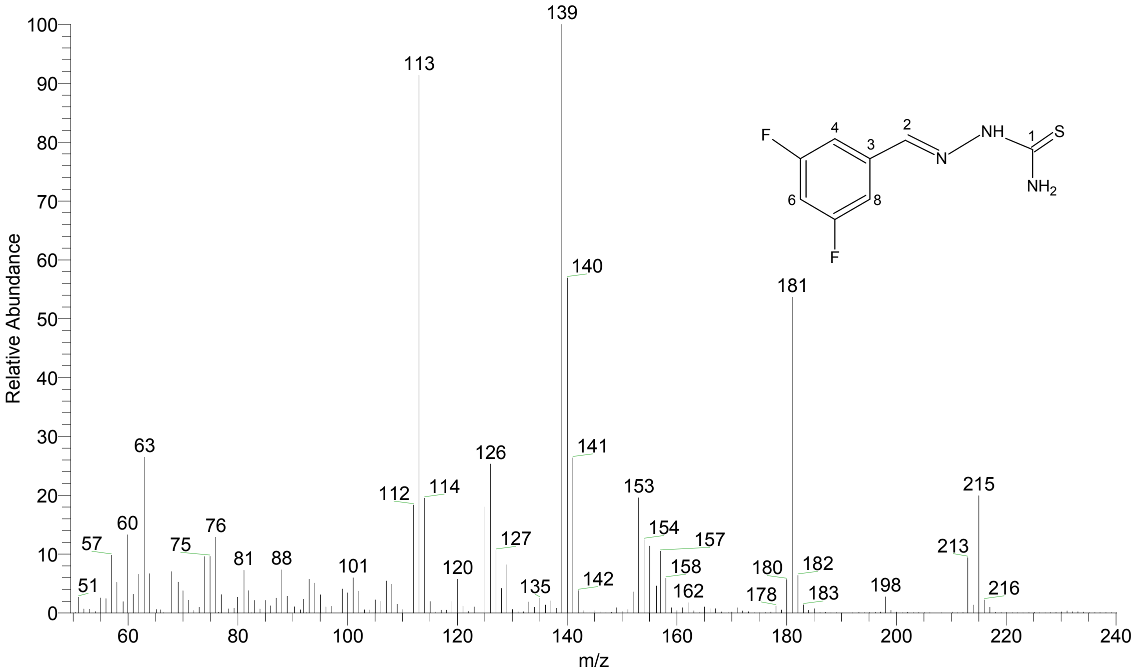

2-(3,5-Difluorobenzylidene)hydrazine-1-carbothioamide (**5**)

Figure S23. ^{1}H-NMR spectrum.

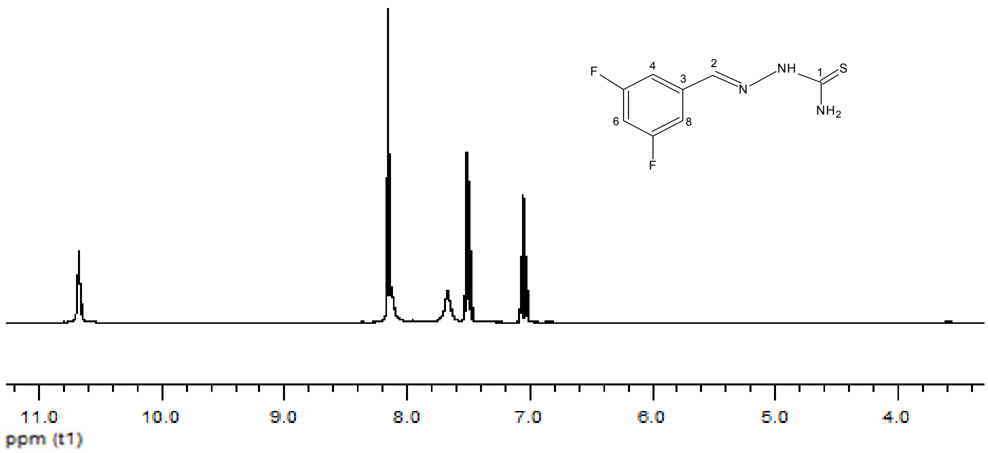

2-(3,5-Difluorobenzylidene)hydrazine-1-carbothioamide (**5**)

Figure S24. ^{13}C-NMR spectrum.

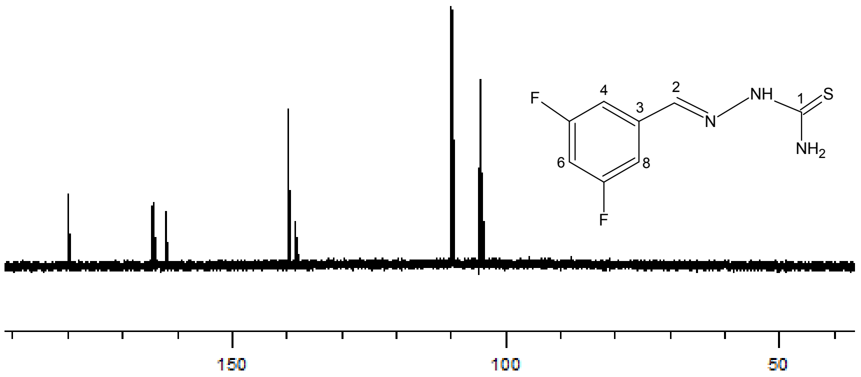

2-(3,5-Difluorobenzylidene)hydrazine-1-carbothioamide (**5**)

Figure S25. ^{19}F-NMR spectrum.

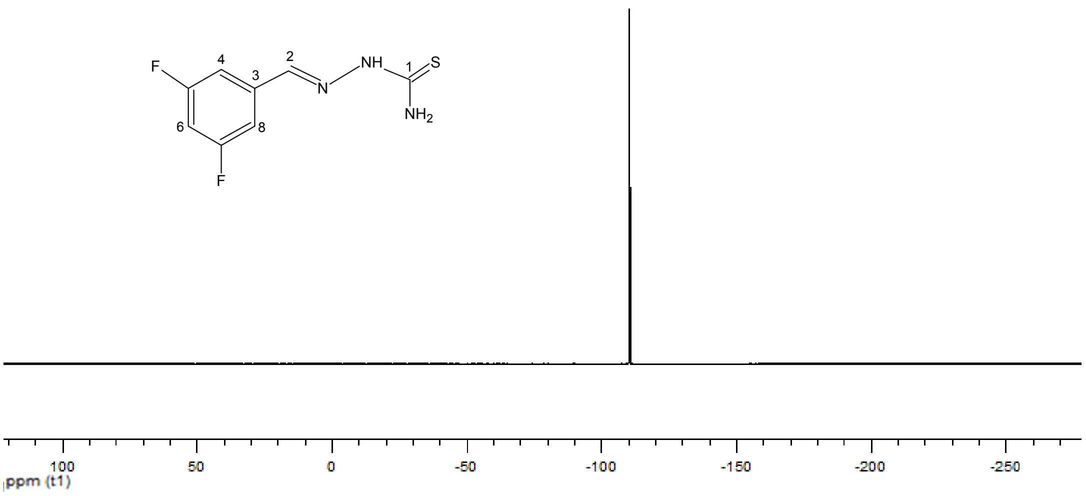

2-(4-Fluoropphenyl)(phenyl)methylene)benzylidene)hydrazine-1-carbothioamide (**6**)

Figure S26. IR spectrum.

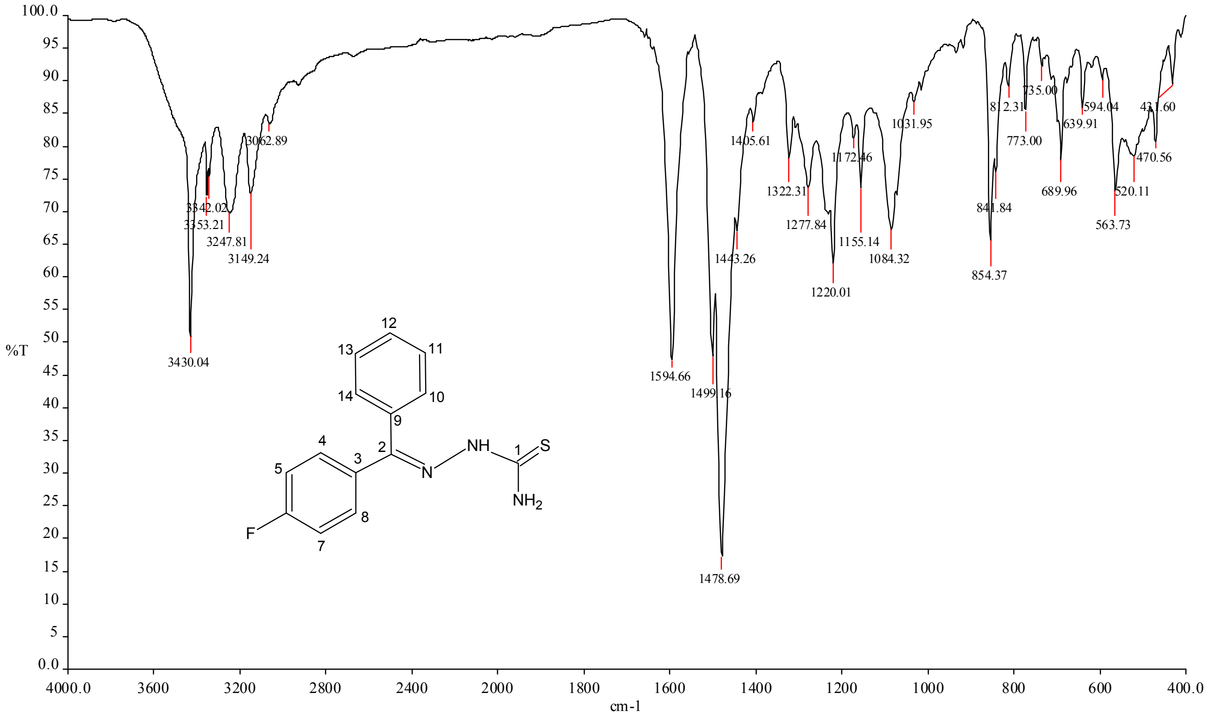

2-(4-Fluoropphenyl)(phenyl)methylene)benzylidene)hydrazine-1-carbothioamide (**6**)

Figure S27. Mass spectrum.

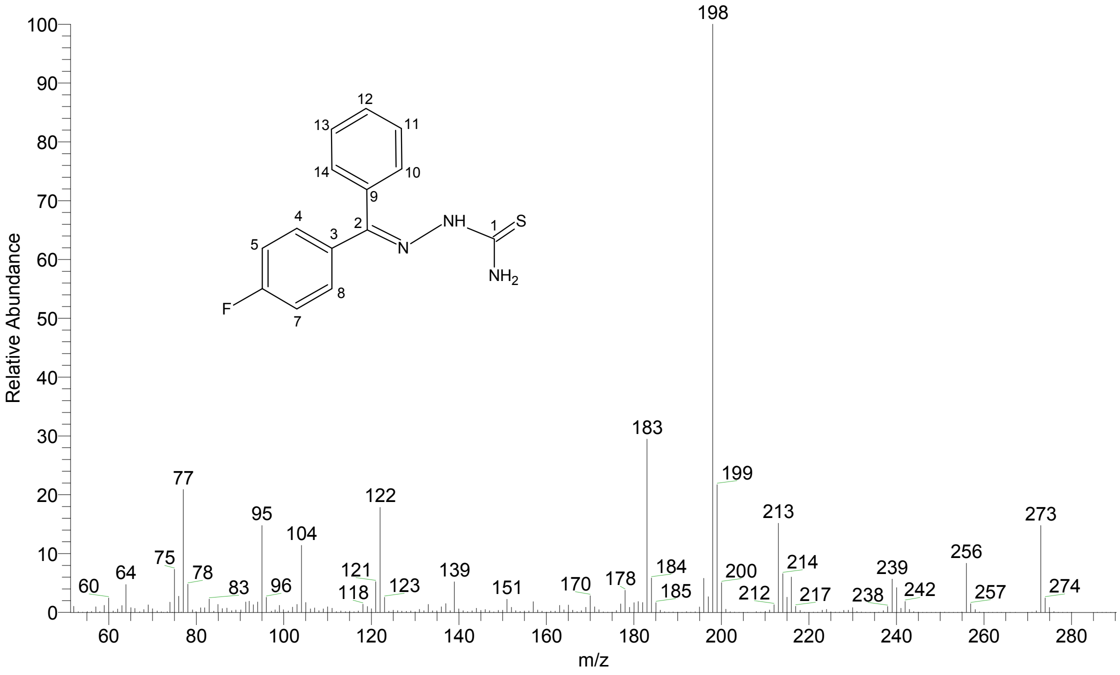

2-(4-Fluoropphenyl)(phenyl)methylene)benzylidene)hydrazine-1-carbothioamide (**6**)

Figure S28. ^{1}H-NMR spectrum.

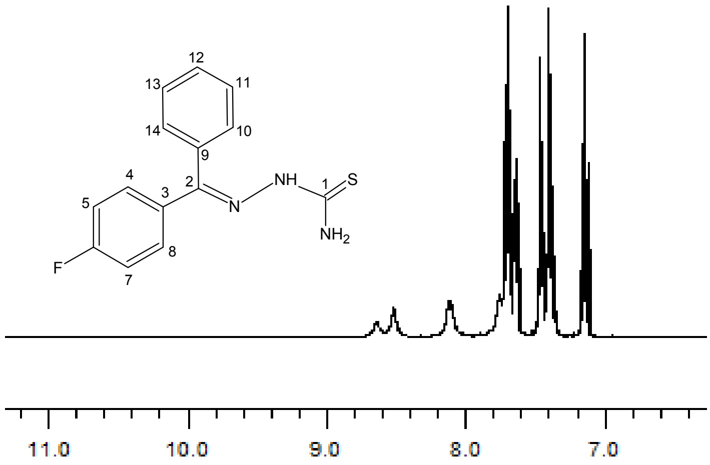

2-(4-Fluoropphenyl)(phenyl)methylene)benzylidene)hydrazine-1-carbothioamide (**6**)

Figure 29. ^{13}C-NMR spectrum

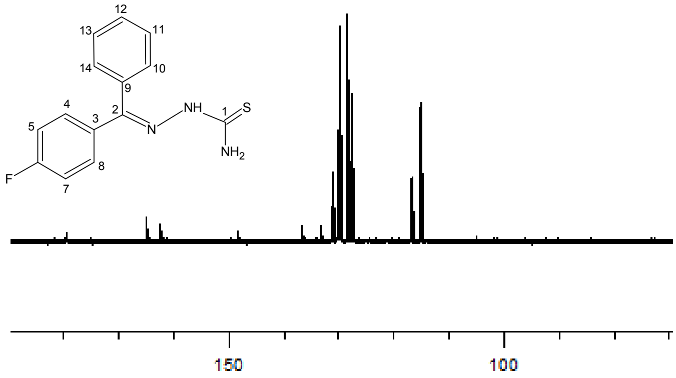

2-(4-Fluoropphenyl)(phenyl)methylene)benzylidene)hydrazine-1-carbothioamide (**6**)

Figure S30. ^{19}F-NMR spectrum.

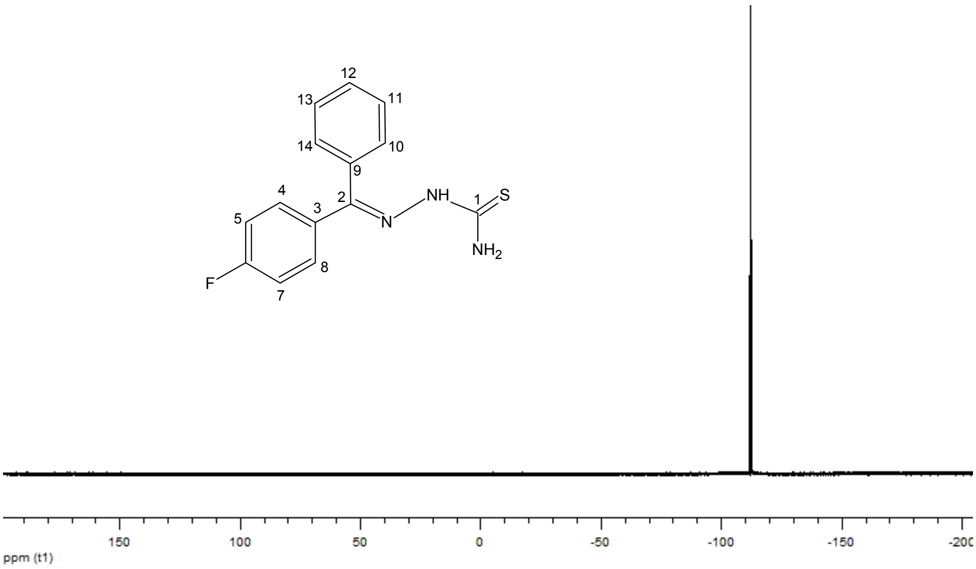

Supplement: Supplementary file 1 [file molecules-18-13111-s001.pdf]
